# The Toll-Like Receptor 4 (TLR4) Variant *rs2149356* and Risk of Gout in European and Polynesian Sample Sets

**DOI:** 10.1371/journal.pone.0147939

**Published:** 2016-01-25

**Authors:** Humaira Rasheed, Cushla McKinney, Lisa K. Stamp, Nicola Dalbeth, Ruth K. Topless, Richard Day, Diluk Kannangara, Kenneth Williams, Malcolm Smith, Matthijs Janssen, Tim L. Jansen, Leo A. Joosten, Timothy R. Radstake, Philip L. Riches, Anne-Kathrin Tausche, Frederic Lioté, Leo Lu, Eli A. Stahl, Hyon K. Choi, Alexander So, Tony R. Merriman

**Affiliations:** 1 Department of Biochemistry, University of Otago, Dunedin, New Zealand; 2 University of Engineering and Technology, Lahore, Pakistan; 3 Department of Medicine, University of Otago, Christchurch, Christchurch, New Zealand; 4 Department of Medicine, University of Auckland, Auckland, New Zealand; 5 School of Medical Sciences, University of New South Wales, Sydney, Australia; 6 Department of Clinical Pharmacology & Toxicology, St Vincent’s Hospital, Sydney, Australia; 7 Department of Medicine, Flinders Medical Centre and Repatriation General Hospital, Adelaide, Australia; 8 Department of Rheumatology, Rijnstate Hospital, Arnhem, The Netherlands; 9 Department of IQ HealthCare, VieCuri Medical Centre, Venlo, The Netherlands; 10 Department of Internal Medicine and Radboud Institute of Molecular Life Science, Radboud University Medical Center, Nijmegen, The Netherlands; 11 Department of Rheumatology and Clinical Immunology, Laboratory of Translational Immunology, Department of Immunology, University Medical Centre Utrecht, Utrecht, The Netherlands; 12 Rheumatic Diseases Unit, Institute of Genetics and Molecular Medicine, University of Edinburgh, Edinburgh, United Kingdom; 13 Department of Rheumatology, University Clinic “Carl-Gustav-Carus”, Dresden, Germany; 14 INSERM, UMR-S 1132, Hospital Lariboisière, Paris, France; 15 University Paris Diderot (UFR de Médecine), Sorbonne Paris Cité, Paris, France; 16 Division of Rheumatology, Allergy, and Immunology, Massachusetts General Hospital, Harvard Medical School, Boston, MA, United States of America; 17 Institute for Genomics and Multiscale Biology, Icahn School of Medicine at Mount Sinai, New York, NY, United States of America; 18 DAL, Service of Rheumatology, Laboratory of Rheumatology, University of Lausanne, CHUV, Nestlé, Lausanne, Switzerland; University of Utah, UNITED STATES

## Abstract

Deposition of crystallized monosodium urate (MSU) in joints as a result of hyperuricemia is a central risk factor for gout. However other factors must exist that control the progression from hyperuricaemia to gout. A previous genetic association study has implicated the toll-like receptor 4 (TLR4) which activates the NLRP3 inflammasome via the nuclear factor-κB signaling pathway upon stimulation by MSU crystals. The T-allele of single nucleotide polymorphism *rs2149356* in *TLR4* is a risk factor associated with gout in a Chinese study. Our aim was to replicate this observation in participants of European and New Zealand Polynesian (Māori and Pacific) ancestry. A total of 2250 clinically-ascertained prevalent gout cases and 13925 controls were used. Non-clinically-ascertained incident gout cases and controls from the Health Professional Follow-up (HPFS) and Nurses Health Studies (NHS) were also used. Genotypes were derived from genome-wide genotype data or directly obtained using Taqman. Logistic regression analysis was done including age, sex, diuretic exposure and ancestry as covariates as appropriate. The T-allele increased the risk of gout in the clinically-ascertained European samples (OR = 1.12, *P* = 0.012) and decreased the risk of gout in Polynesians (OR = 0.80, *P* = 0.011). There was no evidence for association in the HPFS or NHS sample sets. In conclusion *TLR4* SNP *rs2143956* associates with gout risk in prevalent clinically-ascertained gout in Europeans, in a direction consistent with previously published results in Han Chinese. However, with an opposite direction of association in Polynesians and no evidence for association in a non-clinically-ascertained incident gout cohort this variant should be analysed in other international gout genetic data sets to determine if there is genuine evidence for association.

## Introduction

Deposition of crystallized monosodium urate (MSU) in joints as a result of hyperuricemia is a central risk factor for gout. Genome-wide association studies of serum urate have confirmed uric acid transporters SLC2A9 and ABCG2 as important loci, that are also associated with gout, with weaker effects from other uric acid transporters [1–3]. Given that many individuals with hyperuricaemia do not develop gout, other factors must exist controlling the progression from hyperuricaemia to gout [4]. However, very little is known about the genetic basis of the progression from hyperuricaemia to MSU crystal deposition to symptomatic gout [5]. The auto-inflammatory nature of gout involves the activation of the innate immune response by MSU crystals. A central pathway is primability and actual activation of the NLRP3 inflammasome and subsequent release of mature interleukin-1β [6, 7]. An important question is whether or not genetic regulation of this pathway is a risk factor for gout.

Toll-like receptors (TLRs) are transmembrane pattern recognition receptors expressed by innate immune cells that trigger an innate immune response by controlling distinct signaling pathways [8]. In addition to microbial ligands, these receptors also trigger innate immune response against endogenous ligands including MSU crystals [9]. To date ten functional human TLRs (TLR1-10) have been identified [10] of which TLR4 is a prominent member and which has been associated with a number of auto-inflammatory conditions [11]. This receptor activates the NLRP3 inflammasome via the nuclear factor-κB signaling pathway. A *TLR4* single nucleotide polymorphism (SNP; *rs2149356*) has been reported to be associated with gout in a Han Chinese sample set [12], where the TT-genotype was associated with an increased risk of gout (OR = 1.96 [95% CI 1.40–2.74]). The same genotype was associated with increased TLR4 mRNA expression and increased IL1β expression [12]. Our aim was to replicate the genetic association of *rs2149356* with gout in individuals of European and New Zealand Polynesian ancestry.

## Subjects and Methods

### Subjects

Demographic and clinical data are presented in Table 1. All gout cases were clinically ascertained according to the 1977 American Rheumatism Association (ARA) classification criteria [13]. European cases (n = 1614) were recruited from New Zealand (n = 647), by the Eurogout consortium within the European Crystal Network (n = 779) [14] and by the Arthritis Genomics Recruitment Initiative in Australasia (AGRIA; n = 188). These cases were 84.1% male with average age of 63.0 (20–97) years. European non-gouty controls (n = 13005), after exclusion criteria applied as described below, were recruited from NZ (n = 875) and sourced from the Atherosclerosis Risk in Communities (ARIC; n = 8781) and Framingham Heart (FHS; n = 3349 Generation 3 only) studies. The controls were 46.5% male with average age of 50.2 (17–95) years. There were 636 New Zealand Māori and Pacific Island (Polynesian) cases (87.5% male with average age of 49.9 (17–100) years) and 920 controls (46.7% male with average age of 41.1 (17–85) years). Individuals who ever self-reported as having gout or taking urate-lowering medication were excluded from the ARIC and FHS sample sets. The New Zealand Multi-Region Ethics Committee (MEC/105/10/130) and these institutional committees in Europe and Australia granted ethical approval: Research and Ethics Committee, Repatriation General Hospital, South Australia (32/08); Research Ethics Committee, University of New South Wales; Ethikkommission, Technische Universität Dresden (EK 8012012); South East Scotland Research Ethics Committee (04/S1102/41); Commission Cantonale (VD) D'éthique de la Recherche sur l'être Humain, Université de Lausanne; Commissie Mensgebonden Onderzoek regio Arnhem—Nijmegen; Partners Health Care System Institutional Review Board. All subjects gave written informed consent. The Database of Genotype and Phenotype (www.ncbi.nlm.nih.gov/gap) approval number was #834 for accessing data from the ARIC and FHS studies.

**Table 1:** Demographic and clinical data of participants.

	European		NZ Polynesian		HPFS		NHS	
	Control	Gout	Control	Gout	Control	Gout	Control	Gout
Number	13005	1614	920	636	3445	726	6317	351
Male (%)	6049 (46.52)	1332 (84.14)	419 (45.89)	546 (87.50)	3445 (100)	726 (100)	00 (0.00)	00 (0.00)
Age	50.23±10.08	62.95±13.24	41.12±14.45	49.88±13.18	62.02±8.52**	61.73±9.60**	67.99±6.70**	64.95±9.85**
BMI	26.82±5.00 (12491)*	30.04±6.60 (1323)	32.67±7.26 (813)	35.79±7.69 (570)	25.84±3.34**	27.20±3.73**	26.31±5.15**	30.16±6.34**
Serum Urate (mmol/L)	0.336±0.087 (12483)	0.399±0.136 (1118)	0.373±0.088 (680)	0.436±0.119 (484)	-	-	-	-
Gout Duration (Years)	_	15.19±12.56 (1289)	_	13.21±11.09 (548)	_	Incident Cases	_	Incident Cases

All values aside from sex are expressed as mean ± SD.

* For BMI, SU and gout duration, the figure inside the brackets represents the number of subjects with available data.

** Obtained at the time of gout onset among cases and at the mid-point time of cohort follow-up among controls.

A separate data set, previously described in ref [15], of European Caucasian individuals was investigated consisting of 726 incident male cases and 3445 controls from the Health Professionals Follow-up Study (HPFS) and 351 incident female cases and 6317 controls from the Nurses Health Study (NHS) (Table 1). All cases were ascertained according to 1977 ARA classification criteria [13] using a self-administered gout questionnaire as previously described [16]. As summarised in ref [15] evaluation of medical records by two board-certified rheumatologists in a random audit set of 50 HPFS men demonstrated 94% (47/50) concordance of the diagnosis of gout between the self-administered questionnaire and the review of medical records and 91% (51/56) concordance in an audit sample of 56 NHS women.

### Genotyping

Taqman genotyping for rs2149356 was performed for the sample sets excepting ARIC, FHS, HPFS and NHS using a Lightcycler 480 Real-Time Polymerase Chain Reaction System (Roche Applied Science, Indianapolis, USA) in 384-well plates. The FHS cohort had been genotyped by the Affymetrix SNP 5 platform and a custom-designed gene-centric 50K SNP platform and *rs2149356* genotype was imputed using MACH1 v1.0.15 with the HapMap CEU sample set as reference haplotypes. In the ARIC sample set *rs2149356* had been genotyped on the Affymetrix SNP 6 platform. The HPFS and NHS sample sets were genotyped using the Illumina Infinium OmniExpress and genotypes were imputed using MACH (imputation quality Rsq = 0.989). This resulted in N = 10807 non-missing genotype calls (1.5% missing). There was no evidence for departure from Hardy Weinberg equilibrium in any of the sample sets presented in Tables 2 and 3 (*P*_HWE_>0.01).

**Table 2:** *Rs2149356* genotype and association with risk of gout in NZ and Europe sample sets using ARIC and FHS controls.

	Gout	All Controls	Adjusted OR[95%CI]^1^	P
European				
GG	716 (0.444)	6093 (0.469)	1	1
GT	700 (0.434)	5529 (0.425)	1.068 [0.940–1.214]	0.32
TT	198 (0.122)	1383 (0.106)	1.317 [1.082–1.605]	0.006
T	1096 (0.340)	8295 (0.319)	1.122 [1.025–1.227]	0.012
NZ Polynesian				
GG	181 (0.285)	241 (0.262)	1	1
GT	317 (0.498)	437 (0.475)	0.950 [0.710–1.272]	0.73
TT	138 (0.217)	242 (0.263)	0.634 [0.450–0.895]	0.009
T	593 (0.466)	921 (0.501)	0.800 [0.674–0.949]	0.011

1 Adjusted by age, sex and (for Polynesian) STRUCTURE estimate of Polynesian ancestry [17].

**Table 3:** *Rs2149356* genotype and association with risk of gout in the HPFS and NHS sample sets.

Genotype	Total	Gout	Unadjusted RR[95% CI]	P	Adjusted RR^1^ [95% CI]	P
*HPFS*	N = 4171	N = 726				
GG	1920	336	1.00	-	1.00	-
GT	1824	315	0.98 [0.84–1.14]	0.78	0.99 [0.84–1.15]	0.84
TT	427	75	1.03 [0.80–1.32]	0.83	1.05 [0.82–1.35]	0.70
T	2251	390	0.99 [0.85–1.14]	0.85	1.00 [0.86–1.15]	0.94
*NHS*	N = 6668	N = 351				
GG	3137	176	1.00		1.00	-
GT	2901	143	0.88 [0.70–1.10]	0.25	0.89 [0.71–1.11]	0.29
TT	630	32	0.93 [0.64–1.36]	0.71	0.97 [0.66–1.42]	0.87
T	3531	175	0.88 [0.72–1.09]	0.24	0.90 [0.73–1.11]	0.31

1 Adjusted by age and diuretic usage.

### Statistical analysis

All logistic regression analysis was done using Intercooled STATA software version 8.0 (College Station, TX 77845, USA). Allelic and genotypic odds ratios for gout were calculated and all analyses were adjusted by age and sex, with the Polynesian analysis additionally adjusted by an estimate of Polynesian ancestry calculated as previously described in order to account for admixture with other ancestral groups [17]. A threshold of *P* < 0.05 was used to declare nominal statistical significance.

## Results

In European and Polynesian subjects, the T-allele of *rs2149356* was associated with the risk of gout when compared to controls unstratified by urate level, but in an opposing direction of association (Table 2; OR_European_ = 1.12, *P* = 0.012 and OR_Polynesian_ = 0.80, *P* = 0.011). The TT-genotype was also associated with risk of gout when compared to the GG genotype, again in an opposing direction of association (Table 1; OR_European_ = 1.32, *P* = 0.006 and OR_Polynesian_ = 0.63, *P* = 0.009). *Rs2149356* was then tested for association with gout in the HPFS and NHS studies (Table 3). There was no evidence for association in either the allelic or genotypic analyses in either of the studies (*P*≥0.31).

## Discussion

Here we attempted to replicate the previously reported association with gout of *TLR4* variant *rs2149356* in Han Chinese in sample sets of European and Polynesian ancestry. In the NZ and Europeans recruited from Europe the association was replicated but with opposing direction in the Polynesian sample set. A similar opposing direction of association between European and Polynesian is also evident at other urate- and gout-associated loci *PRKAG2* and *HLF* [2]. Why this is the case is not resolved, discussed further below. The third sample set (HPFS and NHS) provided no evidence to support association of *rs2149356* with gout. Two characteristics of this sample set would have reduced chances of detecting association, should genuine association exist. First, only ~90% have clinically-ascertained gout [16]. Second, the sample set are incident gout cases (with minimum age of male cases 40 years and female cases 30 years [16]). Prevalent cases are excluded—these excluded cases would be expected to have an earlier age of onset and a stronger heritable component. On balance we are unable to conclude that the *TLR4* locus to be the first replicated genetic risk factor in gout outside of those that influence gout risk via modulation of serum urate levels. However, given the very strong prior functional candidacy of *TLR4*, we do consider that our study provides evidence consistent with a causal role for *TLR4* in gout.

Regarding the inconsistent direction of association with gout between Polynesian, and Han Chinese and European, the simplest explanation is that *rs2149356* is not the causal variant but in linkage disequilibrium with the causal variant, with a Polynesian-specific ancestral recombination event distinguishing the Polynesian haplotypic background around *rs2149356* from European and Han Chinese, resulting in the other (G) allele of *rs2149356* being on the Polynesian risk haplotype. The Polynesian population could be important in trans-ancestral genetic fine-mapping of the etiological variant in *TLR4* with the variant expected to be in a genomic segment with the same alleles associated with gout between Han Chinese, European and Polynesian. Other possibilities are that this marker is subject to stratification effects that have not been adequately controlled in the Polynesian sample set, that there are non-genetic interactors (e.g. alcohol and sugar-sweetened beverage consumption [18–20]) that are unaccounted for and that would generate inconsistent *rs2149356* main effects or that these are chance findings given the modest level of significance.

SNP *rs2149356* is a common genetic variant of weak effect (OR≤1.4). More than 70% of genetic variants for common phenotypes identified by genome-wide association studies map to regulatory regions of the genome [21]. While it is possible that less common non-synonymous functional variants in *TLR4*, that have been associated with other auto-inflammatory conditions [22, 23], may also associate with gout, it is likely that the effect of *rs2149356* on the risk of gout is via an influence on expression of TLR4, either the amount of TLR4 produced and/or relative levels of isoforms. Direct evidence of this was supplied by Qing et al. [12] who associated the TT-genotype *rs2149356* with increased levels of TLR4 mRNA in peripheral blood mononuclear cells (PBMCs) and increased serum interleukin-1β levels in people with acute gout. Conversely the TT-genotype associated with reduced levels of TLR4 mRNA in PBMCs from people with intercritical gout. These important findings are consistent with the increased risk observed for the TT-genotype for gout. Understanding how the T-allele controls expression of TLR4 will be important for improved molecular understanding of the pathogenic role of the TLR4-pathway in gout. Our data, with an opposing direction of association in Polynesian, suggest that *rs2149356* is not causal but in linkage disequilibrium (LD) with, and a marker for, the causal variant. *Rs2149356* maps to intron 4 of *TLR4* but is in strong LD with two SNPs in the promoter of *TLR4* (*rs2737190*, r^2^ = 0.92 in Europeans and 0.97 in Asians, ‘-2570’ [24]; *rs1927914*, r^2^ = 0.93 in Europeans and 0.97 in Asians, -2026’ [24]). The gout risk allele (T) of *rs2149356* would be present (co-inherited) on a haplotype with the minor allele of each of these promoter SNPs. The minor allele (G) of *rs2737190* is predicted to create a v-Myb transcription factor binding site and the minor allele (G) of *rs1927914* is predicted to remove Oct-1 and C/EBP transcription factor binding sites [24]. When studied individually *in vitro*, the *rs2737190* G allele had no effect, but the *rs1927914* G allele significantly decreased basal expression from the TLR4 promoter and increased response to challenge with a uropathogenic *Escherichia coli* strain [24], which is consistent with the observed expression pattern from Qing et al. [12]. This genetic variant is therefore a candidate functional etiological variant for gout.

In conclusion, *TLR4* SNP *rs2143956* is associated with gout risk in prevalent clinically-ascertained gout in Europeans, in a direction consistent with previously published results in Han Chinese [12]. However, with an opposite direction of association in Polynesians and no evidence for association in a non-clinically-ascertained incident gout cohort this variant should be analysed in other international gout genetic data sets.
